# Zhizhu Kuanzhong Capsule in treating patients with functional dyspepsia postprandial distress syndrome: study protocol for a multicenter, randomized, double-blind, placebo-controlled, parallel-group clinical trial

**DOI:** 10.1186/s13063-022-06396-5

**Published:** 2022-06-02

**Authors:** Mengli Xiao, Linda L. D. Zhong, Wai Ching Lam, Yingpan Zhao, Kok-Ann Gwee, Gerald Holtmann, Jan Tack, Hidekazu Suzuki, Min-Hu Chen, Yinglian Xiao, Xiaohua Hou, Jinsong Liu, Yang Li, Xu-Dong Tang, Fang Lu

**Affiliations:** 1 NMPA Key Laboratory for Clinical Research and Evaluation of Traditional Chinese Medicine and National Clinical Research Center for Chinese Medicine Cardiology, Xiyuan Hospital, China Academy of Chinese Medical Sciences, Beijing, China; 2 School of Chinese Medicine, Hong Kong Baptist University, Hong Kong SAR, China; 3 Department of Gastroenterology, Xiyuan Hospital of China Academy of Chinese Medical Sciences, Beijing, China; 4 Department of Medicine, Yong Loo Lin School of Medicine, National University of Singapore and Gleneagles Hospital, Singapore, Singapore; 5 Faculty of Medicine & Faculty of Health & Behavioural Sciences, University of Queensland, Brisbane, QLD Australia; 6 Department of Gastroenterology & Hepatology, Princess Alexandra Hospital, Brisbane, QLD Australia; 7 Department of Gastroenterology, University Hospitals Leuven, Leuven, Belgium; 8 Division of Gastroenterology and Hepatology, Department of Internal Medicine, Tokai University School of Medicine, Isehara, Kanagawa Japan; 9 Division of Gastroenterology and Hepatology, The First Affiliated Hospital, Sun Yatsen University, Guangzhou, Guangdong China; 10 Division of Gastroenterology, Wuhan Union Hospital, Huazhong Science & Technology University, Wuhan, Hubei China; 11 Center for Applied Statistics and School of Statistics, Renmin University of China, Beijing, China

**Keywords:** Zhizhu Kuanzhong, Functional dyspepsia, Postprandial distress syndrome, Randomized controlled trial

## Abstract

**Background:**

Functional dyspepsia (FD) is one of the most common functional gastrointestinal disorders. Based on the various symptoms present in patients with functional dyspepsia postprandial distress syndrome (FD-PDS), routine agents such as acid suppressants, prokinetic drugs, and centrally acting drugs, offer limited treatment choices with potential side effects. As a preliminary clinical trial showed that the marketed product Zhizhu Kuanzhong Capsule (ZZKZ) can improve symptoms in FD-PDS patients, our study aims to provide further evidence on the clinical efficacy and safety of ZZKZ in the treatment of patients with FD-PDS.

**Methods:**

In this multicenter, randomized, patient- and investigator-blinded, placebo-controlled, parallel-group clinical trial, we will recruit patients with FD-PDS from 18 hospitals in China and Australia. The trial will enroll patients with FD-PDS based on the Rome IV diagnostic criteria. A total of 480 eligible patients will be randomized 1:1 into either ZZKZ or placebo group with 8 weeks of treatment and 4 weeks of follow-up. The primary endpoint will be measured by a self-rated Visual Analogue Score (VAS) for the degree of discomfort with both symptoms of postprandial fullness and early satiation, recorded once a day and 7 days a week. The primary analysis will aim to compare the response rate for FD-PDS VAS score between the groups before and after 8 weeks of treatment with an alpha level of 0.05 (2-sided).

**Discussion:**

This trial aims to strengthen the evidence for the efficacy and safety of ZZKZ, a marketed product, in treating FD-PDS. Compared to the previous clinical trial that targeted FD-PDS, this trial will have an 8-week double-blind treatment period to investigate the effect of long-term mediation through comparison with the placebo group.

**Trial registration:**

ClinicalTrials.gov NCT03825692. Registered on 28 January 2019

**Supplementary Information:**

The online version contains supplementary material available at 10.1186/s13063-022-06396-5.

## Background

Functional dyspepsia (FD) is one of the most common functional gastrointestinal disorders [1]. Patients can experience a range of dyspeptic symptoms, including postprandial fullness, early satiety, mid-epigastric pain, and mid-epigastric burning sensation. According to the symptom pattern, FD can be divided into two subtypes: postprandial distress syndrome (PDS) and epigastric pain syndrome (EPS) [1]. A cross-sectional population survey showed that the subtype distribution was 61% PDS, 18% EPS, and 21% overlapping variant with both syndromes [2]. An Italian epidemiological survey showed that 67.5% of patients diagnosed with FD had PDS, and 48% had EPS [3]. In Rome IV, it was noted that PDS is more common than EPS in clinical practice [4].

PDS originated from gastric motor dysfunction, including impaired gastric accommodation [5, 6], leading to a distal redistribution of a meal and, consequently, antral overload [7]. Based on the various symptoms present in patients with PDS, routine agents such as acid suppressants, prokinetic drugs, and centrally acting drugs offer limited treatment choice with potential side effects [8]. As PDS symptoms are prone to recur, safe and effective medications for long-term use become a critical concern for the patients and clinicians [9, 10].

Zhizhu Kuanzhong Capsule (ZZKZ) (National Medical Products Administration (NMPA) approval number Z20020003), manufactured by Shuangren Pharmaceutical Co., Ltd. of Lonch Group, has been marketed in China for more than 10 years and can be purchased in pharmacies or hospitals. ZZKZ is originated from the ancient document “Synopsis of Prescriptions of the Golden Chamber,” which mainly composed of 4 kinds of Chinese herbs: Rhizoma Atractylodis Macrocephalae, Fructus Aurantii Immaturus, Radix Bupleuri, and Fructus Crataegi. These herbs have long been applied in traditional Chinese Medicine (TCM) to alleviate gastrointestinal symptoms. In a multicenter clinical trial in China which applied Cisapride as control, 403 cases (105 cases in the control group, 196 cases in the treatment group, and 102 cases in the open group) were investigated [11]. The results showed that ZZKZ alone could relieve FD symptoms with an overall response rate of 89.2%. In a double-blind, randomized, placebo-controlled study of 392 subjects in 2017, ZZKZ was found to significantly improve symptoms such as early satiety and postprandial fullness discomfort in patients with FD-PDS [12].

ZZKZ also exhibited therapeutic effects on anxiety and depression through animal experiments and clinical studies. An animal study showed that ZZKZ improved the depressive behaviors of the animals, which the mechanism may be associated with the improvement of 5-HT neuron transmission and the inhibition of stress-induced corticoid secretion [13]. A multicenter clinical study of 202 patients showed that ZZKZ reduced the depression score in patients with FD-PDS and improved the depressive symptoms [14]. Another study showed that in patients with FD accompanied with anxiety and depression, there was no significant difference between the 4-week ZZKZ group and the Domperidone plus Neurostan group in the improvement of anxiety and depression scores [15].

In this trial, a placebo will be selected as the control since placebo design makes the trial easier for researchers to assess the side effects of ZZKZ and reduce biases through comparison of the groups. With evidence from the preliminary clinical trial that investigated the short-term use of ZZKZ [11, 12, 14–16], clinical studies on the effects of long-term ZZKZ use, including the degree of improvement in PDS symptoms, improvement in anxiety and depression, and safety of long-term efficacy, are still lacking. Aiming to consolidate the short-term evidence and explore the effect of long-term use of the medication, we design this trial to evaluate the clinical efficacy and safety of ZZKZ in the treatment of FD-PDS.

### Objectives

The study aims to evaluate the clinical efficacy and safety of the trial drug, ZZKZ, in the treatment of patients with FD-PDS.

## Methods and analysis

### Trial design

**Fig. 1:**
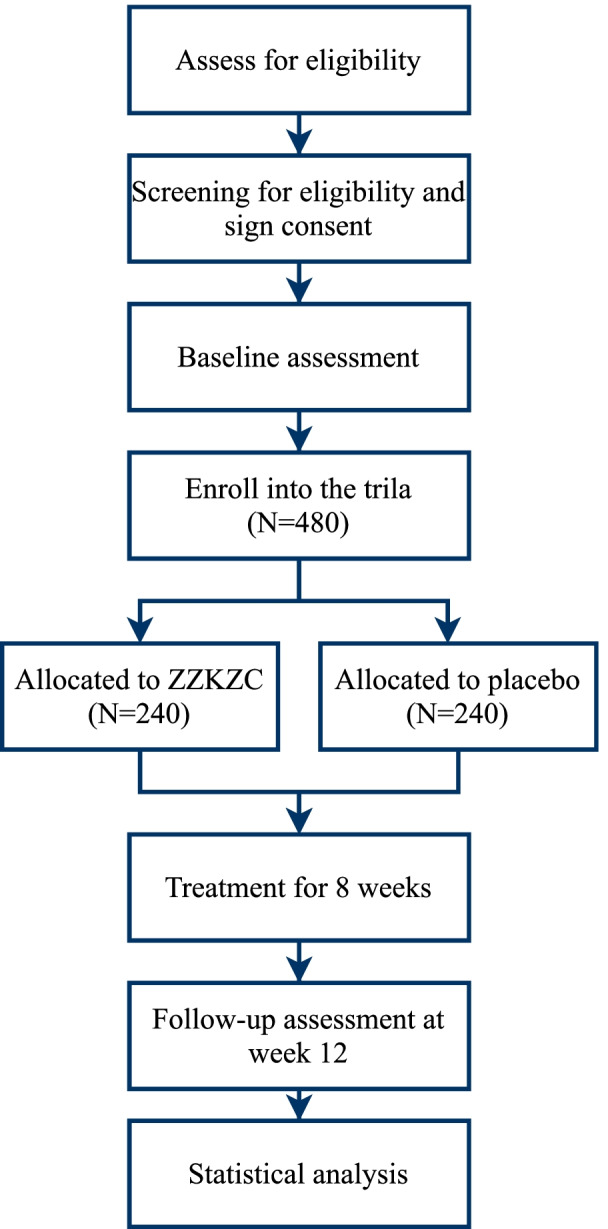
Flow diagram of the trial

This multicenter, randomized, patient- and investigator-blinded, placebo-controlled, parallel-group study will be conducted at 18 hospitals in China and Australia. Patients fulfilling the Rome IV FD-PDS criteria [17] will be recruited primarily through advertisements on hospital websites and outpatient clinics. Before randomization, all patients will be required to provide written informed consent. In response to the COVID-19 pandemic, face-to-face site visits may be replaced by telephone consultations, and study materials may be distributed and returned via mail. The study protocol (version XYYY-V-2.1) has been approved by ethics committees at all participating hospitals following the Declaration of Helsinki and is reported based on SPIRIT (Supplementary file 1) [18]. This trial has been registered on ClinicalTrials.gov (NCT03825692). A flow diagram of the trial is shown in Fig. 1.

### Eligibility criteria

#### Inclusion criteria

(1) Outpatients aged 18–65 years; (2) meeting the Rome IV diagnostic criteria for FD-PDS; (3) able to discontinue prohibited medications that may affect the evaluation of the effectiveness; (4) with Visual Analogue Score (VAS) score ≥ 4 for major symptoms (at least one of postprandial fullness discomfort and early satiety); and (5) informed and voluntarily sign the informed consent form.

#### Exclusion criteria

(1) Gastroscopic findings of gastric cancer, peptic ulcer, erosive gastritis (grade 2 or higher), moderate to severe atrophic gastritis, dysplasia, or other organ gastrointestinal disease; (2) history of abdominal surgery (except for appendectomy and cesarean section); (3) defects in immune function, or using immunosuppressive agents or glucocorticoids within the past 3 months; (4) combined severe heart and lung insufficiency, liver and kidney dysfunction, an endocrine disorder, hematopoietic disorder, hematological tests revealed iron deficiency anemia; (5) severe anxiety or depression; (6) psychotic patients and those with intellectual or language disabilities; (7) pregnancy or breastfeeding; (8) allergy to the ingredients of the investigational drug; (9) drug or alcohol abuse; (10) participated in clinical trials in the past 3 months; (11) been regarded by the investigator as not feasible for this clinical trial.

#### Withdrawal and dropout criteria

Investigators and subjects both can initiate withdrawal, while investigator-initiated withdrawals include (1) if the subject’s condition continues to worsen during the trial, and continuation of enrollment may lead to a serious adverse event; (2) during the trial, the subject experienced certain comorbidities, complications, or special physiological changes; (3) in the trial, subjects’ poor compliance, automatic change of medication, or concomitant use of prohibited medicinal products as specified in the protocol affected the determination of efficacy and safety; (4) adverse events or serious adverse events occur, and the investigators determine it is inappropriate for the subject to continue to receive the test treatment; (5) during the trial, cases were unblinded for various reasons; and subject withdrawals at discretion include (1) the subject perceives the efficacy as poor; (2) intolerance to certain adverse reactions; and (3) whatever the cause, the subject is unwilling to continue to participate in the clinical trial.

All subjects who complete the informed consent form and are deemed eligible to enter the randomized trial through screening, regardless of the reason for withdrawal, will be considered dropout cases as long as they do not complete the observation cycle specified in the protocol. This will include both the subject’s voluntary withdrawal and investigator-initiated withdrawal.

### Randomization and allocation concealment

Stratified-block randomization method by SAS 9.3 statistical software will be used to centrally generate a random number table stratified within each participating hospital according to the number of cases allocated to each participating hospital with a ratio of 1:1 and will be performed by a statistician in a third institution. Parameters of the selected block’s length and the random initial seed will be sealed together as secret data in the blind codes.

### Implementation plan

Each drug will be randomly coded with an identification number according to the random number table and distributed to each site. Each site will further dispense the drug identification numbers according to the order of the subject’s visit and would not be able to alter the numbering arbitrarily.

### Blinding

Both patients and investigators will be blinded to the assigned group. To achieve the blind method, the blind codes of this study which consist of two levels, will be held by the sponsor. The level I blind code will designate whether each drug number will be associated with drug A or drug B, respectively; the level II blind code will designate whether drug A and drug B will be a trial drug or a placebo. According to the statistical analysis plan, the level I blind code will not be revealed until the statistical analysis, which will be carried out by a statistical analyst. After the analyst writes a report, we will hold a concluding meeting, the level II blind code will be revealed for the first time on the spot, and the unblinded staff will sign on the blind code.

Unblinding will be permissible when serious adverse events occur and the actions to protect the safety of the subject must be taken immediately during the study. When urgent unblinding is required, the treating physician shall report to the principal investigator of the site. After approval, the emergency letter sent with the drug will be unpacked, the type of drug administered will be identified, a prompt rescue will be made, and the potential reasons will be clearly explained; the medical record will be signed and dated. Concurrently, the subject will be discontinued from the trial.

### Interventions

The intervention will have a total duration of 8 weeks. Eligible patients will be randomly assigned to receive either the trial drug or a placebo. Their medication will be free for the entire study period.

#### Experimental

ZZKZ (0.43 g per capsule) (Lonch Group Shuangren Pharmaceutical Co., Ltd.; batch number: Z20020003) is composed of the following TCM crude drugs: Rhizoma Atractylodis Macrocephalae (0.16 g, accounting for 37.5%), Fructus Aurantii Immaturus (0.11 g, accounting for 25%), Radix Bupleuri (0.08 g, accounting for 18.75%), and Fructus Crataegi (0.08 g, accounting for 18.75%). The parts of the plant used are listed in Chinese Pharmacopoeia (version 2015). ZZKZ is a standardized product that uses the four dry herbs manufactured as concentrated granules (using 65% ethanol as solvent) and packed into capsules. The ratio of herbal drugs to extract is 2.79:1. The manufacturer keeps the lot numbers of the raw materials with the authentication of raw materials. Qualitative tests, including chromatographic analysis, microscopic identification, thin-layer identification, examinations of moisture, impurities, total ash, heavy metals, harmful elements, and residual sulfur dioxide, have been carried out for ZZKZ, the four herbs by the manufacturer.

#### Placebo

ZZKZ mimics (0.43 g per capsule) (Lonch Group Shuangren Pharmaceutical Co., Ltd.). The placebo does not contain any drug, and starch and microcrystalline cellulose are used as filling agents. The placebo for ZZKZ capsules (0.43 g) contains starch and microcrystalline cellulose in a ratio of 2:1, with an additional amount of an appropriate amount of a food-grade pigment solution, using magnesium stearate as a lubricant (0.5%). Food additives that meet China’s quality standards for pharmaceutical excipients are used for placebo color modulation. The placebo has the same taste, odor, and color as ZZKZ. The dose, method of use, and duration of the investigational products are listed in Table 1.

**Table 1:** Dose and duration of the investigational products

Group	Investigational product	Dose and methods	Duration (weeks)
ZZKZ	ZZKZ	3 capsules at a time, 3 times a day, taken orally 10–15 min before meals	8
Placebo	ZZKZ Mimics	3 capsules at a time, 3 times a day, taken orally 10–15 min before meals	8

### Concomitant drug

Long-term concomitant medication which may have treatment effects on PDS should be avoided:

1. Medicines related to the treatment of PDS should not be administered during the study.
2. Patients taking non-steroidal anti-inflammatory drugs such as aspirin before enrollment may continue taking these drugs without increasing or reducing the dose.
3. Opioid preparations will be prohibited during the trial. If used before enrollment, sedative and hypnotic drugs (e.g., patients with insomnia take diazepam, etc., as needed) and antidepressants can be continued without increasing or decreasing the dose.
4. Some antibiotics that affect gastric motility will be prohibited during the trial: macrolide antimicrobial or azole antifungal.
5. During the trial, acupuncture, massage, cupping, and other TCM therapies related to the treatment of this disease shall not be used.
6. If the participant is eligible by the inclusion criteria but needs to continue taking medicine or to add other drugs or treatment methods for the consideration of comorbid illness or changes of illness state, it will be necessary to record all information to the case report form, including the name of the drug (or other therapies), the dosage, use frequency, and time.

### Outcomes

#### Primary outcome

Participants self-rate on the VAS for the degree of discomfort with both symptoms of postprandial fullness and early satiation, degree of discomfort will be indicated by a 10-cm line marked from 0 (asymptomatic or no discomfort) to 10 (extreme severe or extreme discomfort) [19]. The rating will be made once a day and 7 days a week via a diary card. For VAS scores for postprandial fullness discomfort and early satiety, the integral average for both symptoms over the past week will be evaluated, and a 50% decrease from baseline in the integral average at 8 weeks will be recorded as a response. The proportion of the response at 8 weeks after randomization will be considered the primary efficacy endpoint.

#### Secondary outcomes

##### VAS score of each FD symptom

On the diary cards, subjects will be recorded the VAS of each FD symptom daily, including abdominal distension (mid-epigastric distension or lower abdominal distension), abdominal pain (mid-epigastric pain or lower abdominal pain), epigastric burning sensation, nausea, excessive eructation, heartburn, vomiting, regurgitation, dysphagia, abdominal enlargement, and defecation smoothness [20]. VAS will be scored on a scale of 0–10 which the higher the score, the more severe the sign. The record will be made once a day and 7 days a week. The investigators will use the average VAS scores as the symptom intensity score for the week. The change in each symptom’s score at 8 weeks after randomization relative to the baseline will be evaluated.

##### Overall treatment response rate

The overall treatment efficacy will be evaluated using a 7-point Likert Overall Evaluation Scale [21]. The clinical investigators will ask the subjects the following question weekly: “In the last week, how much have your dyspeptic symptoms been alleviated as compared to pre-treatment?” There will be seven options: (1) the symptoms improved significantly, (2) the symptoms improved, (3) the symptoms improved slightly, (4) the symptoms did not change, (5) the symptoms aggravated somewhat, (6) the symptoms aggravated, and (7) the symptoms aggravated significantly. At the last visit time point of the treatment cycle, patients who selected (1) and (2) will be defined as treatment responders, and those who chose (3) to (7) will be defined as non-responders. The response rates at 8 weeks after randomization between the groups will be compared for differences.

##### Short Form Nepean Dyspepsia Index (SFNDI)

SFNDI is a reliable and valid measure of FD’s quality of life. It has ten items on how subjects’ stomach pain, discomfort, or other epigastric symptoms affected their lives over the last 14 days [21]. Add up the ten items for each of the five subscale scores (range of each subscale 2–10). The SFNDI score changes at 4 and 8 weeks after randomization relative to the baseline will be calculated.

##### Hospital Anxiety and Depression Scale (HAD) score

HAD, mainly used in patients in general hospitals, provides two sets of tests to assess anxiety and depression [22]. Among them, “A” stands for anxiety items, “D” stands for depression items, and each item is scored at four levels. Each of the two sets of items is superimposed to obtain their respective total score. A total score of 0 to 7 indicates normal, 8 to 10 indicates borderline abnormal, and 11 to 21 indicates abnormal. The HAD score changes at 4 weeks and 8 weeks after randomization relative to the baseline will be calculated.

### Safety observation

1. Vital signs: Body temperature, heart rate, blood pressure, and respiration (once at each follow-up visit)
2. Blood routine (RBC, WBC, HGB, PLT, PMN%, and LYM%), urine routine (urine WBC, urine RBC, urine PRO, and urine GLU), stool routine plus fecal occult blood test (FOBT, WBC, and RBC), all this will be tested once before and after treatment respectively, twice in total.
3. Hepatic function (alanine aminotransferase (ALT), aspartate aminotransferase (AST), total bilirubin (TBil), alkaline phosphatase (ALP), and gamma-glutamyl transferase (GGT)) and renal function (serum creatinine (SCr), and blood urea nitrogen (BUN)) will be tested once before and after treatment, twice in total.
4. Electrocardiogram (once before and after treatment, respectively, twice in total)
5. Severity and incidence of adverse events (recorded in detail at any time)

**Fig. 2:**
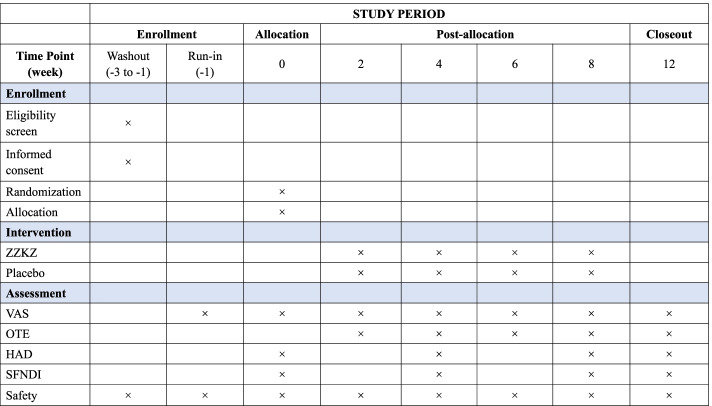
Schedule of the trial

During the trial, each participant will receive safety monitoring. Potential adverse effects will be identified from the data collected by the post-marketing adverse drug reaction monitoring center and the safety data from preliminary trials [23]. All adverse events will be reported to the ethics committee and office of the clinical trial institution. They will review all documented harms during the trial and judge with regard to causality. The enrollment, interventions, and assessment schedule are summarized in Fig. 2.

### Data management

There will be two ways for patients to record their diary cards, using either paper diary cards or an electronic diary (eDiary). Both paper diary cards and eDiary will record the severity of patients’ symptoms related to FD, including postprandial fullness discomfort, early satiety, abdominal distension (mid-epigastric distension or lower abdominal distension), abdominal pain (mid-epigastric pain or lower abdominal pain), epigastric burning sensation, nausea, excessive eructation, heartburn, vomiting, regurgitation, dysphagia, abdominal enlargement, and defecation smoothness. All the investigators will receive training regarding data management and participants’ education for instructions on using the paper diary cards and/or eDiary. For eDiary, in order to ensure that participants can fill out the diary card every day, the system will send a text message to the participants who have not filled out the diary card every night for a reminder. Also, researchers will be able to use the system to check whether participants have filled out the diary card. The original medical records will be kept intact by the site investigators as original documents. The data will be inputted into the eCRF which the database has been established before recruitment. Investigators will be responsible for verifying the accuracy of the data. Data locking will be completed by the data management team, researchers would not be able to modify the data after locked. All research data, including paper and electronic documents, will be kept for at least 5 years after publication. All participant data collected during the trial will be deidentified and available for anyone who wishes to access the data immediately following publication.

A Data and Safety Monitoring Board (DSMB) is set up to review the protocol and the research data. The DSMB members will meet regularly to review the trial data according to ethical and safety standards and monitor the data’s authenticity and completeness based on the study design. The DSMB will also review the trial progress, determine adverse events, and have the authority to decide whether the study will need to end early.

### Quality control and assurance

This trial protocol has been reviewed and revised by experts in gastroenterology, methodology, and statistics. Investigators will have to be trained with a prespecified standard operating procedure before the trial, including eligibility criteria, interventions, details in filling eCRF, assessment of outcomes, and data management. An inspection plan will be designed for quality control, and professionals will be hired to supervise the research process. Moreover, patients will be allowed to use authorized accounts in WeChat to fill out the eCRF to improve compliance. The system will send reminders to patients to fill out the eCRF. In addition, the research assistants and clinical research coordinators will be able to monitor participants’ research records through the researcher management backstage.

### Sample size calculation

The study’s treatment duration will be 8 weeks, with the responder rate of the clinical symptoms of FD-PDS after 8 weeks of treatment being the primary efficacy endpoint. To control the overall false-positive error in the trial and to ensure the power of the test, the study will adopt the following assumptions: alpha level of 0.05 (2-sided) and power of 0.8, with the ratio of the number of cases in the ZZKZ group and the placebo group as 1:1. According to the results of a multicenter clinical study of ZZKZ completed by Professor Chen Minhu in the First Affiliated Hospital of Sun Yat-sen University, with the cooperation of 16 domestic hospitals, the responder rate for clinical symptoms of FD-PDS has been set 54% with the ZZKZ and 38% for the placebo [16]. Calculation of sample size indicates that 187 cases will be required in the ZZKZ and placebo groups respectively. Assuming a dropout rate of 20%, a total of 480 cases will be required for our study.

### Statistical analysis

Statistical analysis will be performed using SAS 9.3 software by an independent statistician. All statistical tests will use a two-sided test, *P* ≤ 0.05 will be regarded as significant. A 95% confidence interval will be used. Based on the intention-to-treat principle, the missing data will be replaced by the last data (i.e., the missing efficacy data will be replaced by the efficacy data of the previous follow-up). The efficacy analysis will be mainly based on a full analysis set, and the central effect will be considered. The primary efficacy endpoint will be tested for superiority. The efficacy between groups will be compared using the Cochran-Mantel-Haenszel-*χ*^2^ test with and without regard to center stratification, and a 95% confidence interval of the difference between groups will be calculated. Method repetitive measure analysis of variance will be used to analyze the secondary outcome of VAS scores at different time points between groups. Sub-analysis will be carried out to review whether there is a difference in the effect of ZZKZ according to demographical data across countries. The safety analysis will be mainly based on descriptive statistics. The adverse events that occur in this trial will be described in a list, and the incidences will be compared using Fisher’s exact probability test.

### Ethics and dissemination

Institutional ethics approvals for the trial have been obtained from the ethics committee of Xiyuan Hospital and all other participating institutions (Supplementary file 3). This study’s purpose and potential risks will be fully explained to the participants. All participants must provide written informed consent before participating in this study. The model consent form is attached (Supplementary file 2).

Practitioners in gastroenterology who have been authorized as investigators will obtain the consent of potential trial participants. The investigators will explain the study procedures to the potential participants in a language and text that they can understand. After the potential participants have thoroughly read and understood the informed consent form, the informed consent form will be signed and dated by the patient if they agree to participate.

After the patient has signed the informed consent form, relevant personal information will be collected and a subject screening number will be assigned. All information about the patient, including his/her identity, medical history, and illness, will be kept strictly confidential by the researcher. Access to the records will be restricted to authorized investigators and ethics committees only. In all the documents submitted to the sponsor, only the subject screening number for the clinical study can be used to identify the clinical study subject, without indicating the subject’s name and hospital reference number. The subjects’ names will not appear in any public information or reports related to the study.

### Plans for protocol amendments

In case it is necessary to modify the study protocol after the trial has begun, the principal investigator and sponsor will reach a consensus and resubmit the modified protocol to the ethics committee. After obtaining the approval, the modified protocol shall be uploaded to the trial registry and submitted to the researchers in other sub-centers for approval by the ethics committees. Only with the support of the local ethics committee can further research be carried out. Patients participating in the study will be informed if their benefits are involved.

## Discussion

FD-PDS is prone to recur, which seriously affects patients’ quality of life, and brings a huge demand for medical resources [24]. Complying with good clinical practice guidelines and having been examined in clinical trials [23], our study aims to assess and compare the effects of ZZKZ with placebo for treatment of FD-PDS in China and Australia as an attempt to observe the effects across different populations. Compared to the previous clinical trial that targeted FD-PDS [16, 25, 26], we will set up an 8-week patient- and investigator-blinded treatment period to investigate the effect of long-term mediation compared with the placebo group.

We will focus on the patient-reported outcomes to capture the patients’ illness experience in a structured format as a reflection of the real-world clinical situation [27]. The use of a daily diary has the advantages of consecutive recording of disease activity and allowing average values by repeated measurements. Evaluation of the VAS of each FD symptom through daily measurement is considered the most reliable tool to evaluate symptom pattern and severity. However, filling out diary cards daily could be a big challenge for maintaining high compliance of the participants.

FD-PDS is associated with mental stress. Previous animal studies have shown that ZZKZ has an antidepressant effect that can relieve depressant behaviors [12]. To investigate the effect in patients with FD-PDS, HAD will be adopted in this clinical trial to observe the psychological changes of the participants. Moreover, since studies have shown that symptoms may have an inverse relationship with the severity of *H. pylori*-associated inflammation and oxidative damage in patients with FD [28, 29], further studies can consider investigating the effect of ZZKZ in *Helicobacter pylori*-positive patients. TCM has unique theoretical and practical methods in treating diseases, and treatment based on syndrome differentiation is the basic principle of disease recognition and treatment in TCM [30]. As medical records, TCM symptoms (gastrointestinal symptoms, mood, urine, feces, tongue texture, tongue coating, etc.) of the participants will be collected in this study.

There are some limitations in this study. Firstly, ZZKZ is composed of 4 TCM ingredients, and its complex treatment mechanisms will require further investigation. Second, although the current study will be conducted in two countries, the majority of the study’s sub-centers are in China, thus limiting the generalizability of the results. Thirdly, because of the COVID-19 pandemic, some face-to-face site visits will be replaced by telephone consultations, which may create challenges for the collection of data. Lastly, although biomarker provides supporting evidence for the clinical efficacy of FGID treatment, we would not use biomarkers to evaluate the efficacy of the drugs in this study, which leads to a research gap in the explanation of the clinical results from the aspect of biological indicators [26].

## Trial status

The trial has been recruiting patients since Oct 24, 2019. The recruitment will be completed before December 2022.

## Supplementary Information

**Additional file 1.** SPIRIT checklist.

**Additional file 2.** Informed consent form (version XYYY-V-2.1).

**Additional file 3.** Names of all ethics committees and approval reference numbers of all participating institutions.

## Data Availability

All individual participant data collected during the trial will be deidentified and available for anyone who wishes to access the data immediately following publication.

## Abbreviations

FD: Functional dyspepsia
ZZKZ: Zhizhu Kuanzhong Capsule
PDS: Postprandial distress syndrome
EPS: Epigastric pain syndrome
NMPA: National Medical Products Administration
TCM: Traditional Chinese Medicine
VAS: Visual Analogue Score
SFNDI: Short Form Nepean Dyspepsia Index
HAD: Hospital Anxiety and Depression Scale
ALT: Alanine aminotransferase
AST: Aspartate aminotransferase
TBil: Total bilirubin
ALP: Alkaline phosphatase
GGT: Gamma-glutamyl transferase
SCr: Serum creatinine
BUN: Blood urea nitrogen
eDiary: Electronic diary
DSMB: Data and Safety Monitoring Board
