# Supplementary material for: Zhizhu Kuanzhong Capsule in treating patients with functional dyspepsia postprandial distress syndrome: study protocol for a multicenter, randomized, double-blind, placebo-controlled, parallel-group clinical trial
Source: Trials. 2022 Jun 2;23:454. doi: 10.1186/s13063-022-06396-5 (PMC9161179; doi:10.1186/s13063-022-06396-5)
Supplement: Supplementary file 2 — Additional file 2. Informed consent form (version XYYY-V-2.1). [file 13063_2022_6396_MOESM2_ESM.pdf]

Treatment of postprandial discomfort syndrome of functional dyspepsia with Zhizhu Kuanzhong capsule

Clinical Study Informed Consent Form

Informed Consent Page

Dear Patients: Hello!

Your doctor has diagnosed you with functional dyspepsia postprandial discomfort syndrome. You are welcome to voluntarily participate in the multi-center, randomized, double-blind, placebo-controlled clinical study of Zhishu Kuanzhong Capsule in the treatment of postprandial discomfort syndrome of functional dyspepsia, and we would like to express sincere thanks for your participation. Before deciding to participate or nor, you need to understand the purpose of the trial, the investigational product, possible risks to you, what you are expected to do in the trial, and your rights as a subject. Please read this Participant Information Sheet carefully.

1. Study background

Functional dyspepsia is a common functional gastrointestinal disease. Patients present with a series of dyspeptic symptoms such as upper abdominal pain, upper abdominal burning sensation, postprandial fullness, and early satiety. According to the clinical manifestations, patients can be divided into two subtypes: postprandial discomfort syndrome and upper abdominal pain syndrome. Its pathogenesis may be related to gastroduodenal motor dysfunction, visceral hypersensitivity, increased gastric acid secretion, and mental and psychological factors. The available clinical study evidence shows that the efficacy of drugs for gastric acid suppression, gastrointestinal motility improvement, and eradication of Helicobacter pylori infection in patients with functional dyspepsia is limited, and a significant number of patients still have no improvement in symptoms after using the above drugs. At the same time, patients with functional dyspepsia are prone to recurrent symptoms. Such patients often need long-term medication, and long-term use of the above drugs may produce corresponding side effects. Therefore, the selection of drugs with better clinical efficacy and less side effects is an urgent problem to be solved in clinical treatment. Current clinical research data show that traditional Chinese medicine can significantly alleviate the clinical symptoms of patients, with special advantages and good clinical application prospects in the treatment.

Zhizhu Kuanzhong Capsules are manufactured by Lonch Group, Shuangren Pharmaceutical Co., Ltd (GuoYaoZhunZhi Z20020003) and have been marketed for more than ten years. This prescription originates from the traditional classic prescription "Zhizhu Decoction" in Zhang Zhongjing's "Synopsis of Golden Chamber" and the classic prescription Zhizhu Pills in "Differentiation on Endogenous and Exogenous Diseases". Zhizhu Kuanzhong Capsule is mainly composed of the following 4 kinds of Chinese herbs: Atractylodes tonifies spleen and helps transport; Fructus Aurantii lowers Qi and reduces stagnation, eliminates cramps and swelling; Bupleurum not only rises the fresh Qi in spleen and stomach, and disperses the stagnation of liver qi; and hawthorn helps digestion and tonifies spleen. These four kinds of herbs coordinate with each other to treat spleen deficiency, qi stagnation, liver-stomach disharmony as well as stomach duct and abdomen fullness.

Zhizhu Kuanzhong Capsule has been widely used in the treatment of functional dyspepsia. In a multi-center clinical trial in China, 403 cases (105 cases in the control group, 196 cases in the treatment group and 102 cases in the open group) were observed with cisapride as the control. The results showed that Zhizhu Kuanzhong Capsule had a significant effect in the treatment of functional dyspepsia with a total effective rate of 89.2%. In 2017, a placebo-controlled RCT study included a total of 392 subjects. The study results showed that Zhizhu Kuanzhong Capsule could significantly improve the symptoms of early satiety and postprandial fullness discomfort in patients with functional dyspepsia postprandial discomfort syndrome compared with placebo (50% vs 30%).

Modern pharmacological studies have shown that Fructus Aurantii Immaturus can relieve small intestinal spasm caused by acetylcholine or barium chloride and can increase the intestinal contraction rhythm. Atractylodes Rhizome can significantly antagonize the intestinal spasm caused by acetylcholine or barium chloride, promote gastrointestinal conduction, enhance gastrointestinal motility and regulate gastrointestinal function. Zhishu Kuanzhong Capsule is a new type of pure Chinese medicine gastrointestinal function regulator. Initial clinical trials have shown that it has a good effect on FD patients. The effect is to stimulate the cells to release motilin to promote gastric emptying through the Fructus Aurantii Immaturus, so it can significantly improve the symptoms of fullness, epigastric pain, belching and nausea and vomiting.

2. Study introduction

This study is a post-marketing international multicenter clinical study and one of the main research contents of the project "Demonstration Study of International Cooperation in Traditional Chinese Medicine for the ‘One Belt One Road' Country", which is jointly funded by the Special Fund for Modernization of Traditional Chinese Medicine Research of the National Key R & D Program in the 13th Five-Year Plan and Lonch Group, Shuangren Pharmaceutical Co., Ltd. and has been approved by the Ethics Committee of Xiyuan Hospital, China Academy of Chinese Medical Sciences.

The purpose of this study is to evaluate the clinical efficacy and safety of Zhishu Kuanzhong Capsule in the treatment of postprandial discomfort syndrome of functional dyspepsia.

This study is a multicenter, randomized, controlled clinical study, which is divided into test group and placebo group. If you meet the inclusion criteria and agree to participate in the study, you will be randomly assigned to one of the two groups. You have an equal chance of entering the trial and the placebo group. Neither your doctor nor you will know your group assignment and medication. The placebo effect on functional dyspepsia has been reported to range from 13% to 73%.

3. Who is suitable for the study

If you suffer from functional dyspepsia and postprandial discomfort syndrome accompanied by some degree of postprandial discomfort and early satiety symptoms, aged 18 ~ 65 years old, we will sincerely invite you to participate in this study.

4. Who should not participate in the study

You will receive a comprehensive medical examination before joining the study. We recommend that patients with the following conditions should not participate in this study: Abnormalities were found by gastroscopy and laboratory tests, with evidence of overt gastrointestinal bleeding, gastrointestinal inflammation (ulcer, erosion, bleeding), including melena, hematemesis, palpable abdominal mass; severe anxiety and depression;

history of gastric surgery; immune dysfunction (such as leukemia, cancer patients, etc.), or use of immunosuppressive agents or glucocorticoids in the past 3 months; severe heart and lung dysfunction, liver and kidney function, endocrine system, hematopoietic system abnormalities, hematological examination indicating the presence of iron deficiency anemia; psychiatric patients and mental and language disorders; pregnant (women with positive results in childbearing age pregnancy test) or lactating women, women with recent fertility plan; allergic to the composition of this preparation; participate in the clinical trial in last 3 months; suspected or indeed alcohol and drug abuse history; those who researchers believe not suitable to participate in clinical trials.

5. Overall process

This study needs to take up some time for you to cooperate. You need to go through at least 1 week of run-in period, 8 weeks of double-blind medication treatment period and 4 weeks of withdrawal follow-up period. If you are taking the drugs prohibited in this study before screening, you need to go through 2 weeks of washout period.

(1) Before you are enrolled in the study, you need to sign an informed consent form and complete relevant medical examinations.

(2) Study methods

Zhizhu Kuanzhong Capsule (or Zhizhu Kuanzhong Placebo Capsule), 3 capsules one time, 3 times a day, oral administration 10-15 minutes before meals.

The excipients of Zhizhu Kuanzhong placebo capsule are the same as those of the test drug, basically consistent with the taste, odor and color of the test drug, and the colorant complies with the Quality Standard for Pharmaceutical Excipients of China.

Symptoms of functional dyspepsia are usually related to poor eating habits and changes in mental status. Even with no drug intervention, there is a tendency for natural remission. There is no recognized effective drug for the treatment of functional dyspepsia. During the study, you need to adjust your diet and life style:

During the study, try to keep the eating habits relatively consistent and the diet should be light. You should give up smoking, alcohol, coffee, strong tea, spicy, irritating and cold foods and avoid high-fat, hard and greasy foods and indigestible foods, (such as carbonated beverages, coarse grains, glutinous rice, onions, potatoes, sweet potatoes and sweets) etc. Three meals should be regular and with moderate intake. Keep a regular diet. Regulate life style, ensure adequate sleep, maintain a good attitude, avoid emotional and sullen, appropriate participation in sports and physical labor within your capacity.

(3) Study observation time points and relevant medical examinations

Pre-inclusion check/washout (-3 weeks to -1 week):	Vital signs, clinical symptoms, three major routine indicators of blood, urine, stool, liver and kidney function, urine pregnancy (women of childbearing age), electrocardiogram, gastroscopy, Helicobacter pylori and other tests, if necessary, conduct pathological examination; those who taking banned drugs before screening need to be washed out for 2 weeks; eligible candidates enter the 1-week run-in period.
- 7-Day Run-in: (-1 week prior to enrollment)	Record of clinical symptoms such as postprandial discomfort of functional dyspepsia.

Enrolled on Day 0: (Baseline)	Complete the vital signs, clinical symptoms, and quality of life questionnaires.
Re-examination on Day 14: (Week 2 after first dose)	Examination of vital signs, clinical symptoms, etc.
Re-examination on Day 28: (Week 4 after first dose)	Examination of vital signs, clinical symptoms, etc.
Day 42 Follow-up Phone Call: (Week 6 after first dose)	Record of clinical symptoms.
Return visit on Day 56: (8th week after administration)	Vital signs, clinical symptoms, blood, urine, stool three routine, liver and kidney function, ECG and other examinations.
Re-examination on Day 84: (Week 4 of withdrawal follow-up period)	Symptoms such as postprandial discomfort of functional dyspepsia.
Call or return visit at any time:	When the degree of abdominal distension and abdominal pain was intolerable, call the doctor to get instruction on concomitant medication.

During the trial, you need to record the onset or remission of your dyspeptic symptoms every day, and visit the doctor according to the follow-up time agreed with you and your needs. Your records and follow-up are important because the doctor will determine whether the treatment you receive actually works.

Please follow the instructions of your doctor and record the medication in a timely and objective manner after each administration. Also at each re-examination, inform your doctor about the medications you must continue to take during the trial due to other diseases.

During the trial in addition to the medication specified in the protocol:

(1) Do not add Chinese and Western medicines related to the treatment of this disease, such as: Gastric motility drugs, gastric mucosal protective agents, antacids, H2 receptor antagonists, proton pump inhibitors, digestive aids, antiemetics, anticholinergics, and proprietary Chinese medicines that can improve dyspeptic symptoms are clearly indicated on the package insert.

(2) Certain antibiotics that affect gastric motility: Macrolide antibacterial /azole antifungal;

(3) Do not use acupuncture, massage, cupping and other traditional Chinese medicine treatment related to the disease;

(4) Opium preparations were banned during the trial, and use sedative hypnotics (such as diazepam and other drugs taken as needed by insomnia patients) before enrollment and continue to use it on the premise of not increasing or decreasing the dose.

(5) Patients using non-steroidal anti-inflammatory drugs such as aspirin before enrollment may be continue to be used on the premise of not increasing or decreasing the dose.

If you need other treatment, please contact your doctor beforehand. Medication taken should be truthfully

recorded in the subject diary.

6、Your rights and interests

(1) The researchers will introduce the medicines under test and the test arrangements, you can choose to participate the project or not as you wish. The researchers will report you of all the events related with you, so that you can decide to continue to participate the project in any time. If you have any questions, you can call or directly consult the researchers. You will enjoy good treatment service during the research. You may choose to reject the research, or withdraw from the research at any time during the research, this will not affect the relationship between you and the doctor, your treatment, medical remuneration, or rights or interests, cause other profit losses, or make you suffer discrimination or unfair treatment.

You don't have to participate the research for treating your disease. Other western medicine or traditional Chinese medicine treatments can also be used.

You will receive good medical care and professional guidance on your disease and treatment during the study. If the disease is not controlled and continues to worsen during the study, you may choose to withdraw from the trial and enter routine clinical treatment according to the normal treatment procedures;

If you withdraw from the research halfway due to the medicines, please tell the doctors for any disease change, and accept corresponding physical examination and physical and chemical inspection, this is very beneficial to your self and the whole research.

(2) You and the society may benefit from the test in the future. This benefit includes that your condition may be improved, and you will receive the investigational drug provided by the study group free of charge during the trial as well as the laboratory tests related to this trial, including a gastroscopy and HP examination during the screening period, and blood routine, blood biochemistry, urine routine, stool routine and ECG examination after 8 weeks of screening and double-blind treatment, respectively. Traffic allowance of 300 yuan is available after you have completed all visits, and you will enter routine clinical care according to your condition.

(3) If there is any new information affecting the subject's continued participation in the trial, your doctor will inform you or your legal representative in a timely manner.

(4) If you accept the treatments and inspections for other diseases at the same time, it will not be in the range of free of charge.

7. Your obligations

Participate the research willingly, and sign Informed Consent Form before the test.

Obey the program of clinical trials, and follow the uniform arrangements by the researchers. Cooperate the researchers in completing the testing tasks.

Other medicines are prohibited during the test, and the researchers shall be informed if such medicines are required.

8. Potential adverse reactions and safety measures

All the medicines used may have side effects. During participation in this study, if possible adverse reactions occur, please contact your doctor in charge in time, and he/she will take corresponding medical measures. In the event of any injury occurring during the course of the trial as a result of the use of the trial medication or in compliance with the procedures specified in the trial, this Study Partner, Lonch Group,

Shuangren Pharmaceutical Co., Ltd., will be responsible for the medical expenses incurred in the diagnosis and treatment of the injury and the corresponding compensation.

9. Reasons for terminating your participation in the trial

(1) The investigator believes that your safety may be compromised.

(2) If you experience a poor response or some side effects that are intolerable.

(3) You do not take your medicine as directed by your doctor.

(4) Serious adverse reactions occurred during the study.

(5) The clinical trial was cancelled by CFDA/relevant national departments.

In case of the above situations, the investigator has the right to terminate your participation in the trial without your consent.

10. Confidentiality

The confidentiality of all your information, including your identity, medical history, conditions, physical examination and laboratory test results, etc., shall be strictly kept within the scope permitted by law. Only the researchers authorized, the Ethics Committee, and the Project Approval Department can review your records, and regulatory authorities of CFDA will be allowed to review your medical history related to this research to confirm the authenticity and accuracy of the materials collected for this research, but your detailed personal information will not be involved. Your name and personnel information will not appear in published materials or reports related to this research. We will make every effort to keep your information confidential within the law.

11. Publication

Whatever the results may be, we will try our best to publish the research results.

12. What needs to be done if you participate in the study?

(1) Provide accurate information on past and current medical history.

(2) Tell the study doctor about any health problems you have during the study.

(3) Tell the study doctor about any new medicines, medicines or herbs you take during the study.

(4) You should not take any medications or use other treatments than those specified in the protocol unless approved by your study doctor. Take trial medication as directed and visit as required.

(5) Return unused trial medication and all empty packages to the study doctor at each required visit.

(6) Record subject diary cards and carry them at each visit.

(7) Store the study drug at room temperature, keep it out of the reach of children, and do not give the study drug to others.

(8) Do not participate in other medical research at the same time.

(9) Follow the study staff and study doctor's instructions.

(10) you can inquire at any time anything is not clear to you.

Thank you for reading the above information. If you decide to participate in the clinical study, please tell your doctor who will arrange everything for you about the clinical study.

Please keep this information.

Signature Page of Informed Consent Form

Trial Name: A Multi-center, Randomized, Double-Blind, Placebo-Controlled Parallel Group Clinical Research of Zhizhu Kuanzhong Capsule in Treating Patients with Functional Dyspepsia-Postprandial Distress Syndrome

Responsible unit of the project: Xiyuan Hospital, China Academy of Chinese Medical Sciences

Project partner: Lonch Group, Shuangren Pharmaceutical Co., Ltd.

Subject number /Drug approval number: 2017YFC1703703/GuoYaoZhunZi Z19990069

Statement of Consent

I have read the introduction of the research above, and had the chance of discussing with the doctors on the research and proposing my questions. All the questions I proposed have been clearly answered, and up to my satisfaction.

I am aware of the potential risks and profits of participating the research. I know that one shall participate the research at free will, I confirm that I have sufficient time to consider, and understand:

- I can ask the doctors for more information at any time.
- I can withdraw from the research at any time, this will not affect my treatment and equities, or make me suffer discrimination or unfair treatment.

I also understand that if I withdraw from the research halfway, specially due to the medicines, I will tell the doctors for any disease change, and accept corresponding physical examination and physical and chemical inspection, which is beneficial to my self and the whole research.

If I need any other medical treatment due to the disease change, I will ask for the doctor's advice in advance, or tell the doctor the truth afterward.

I will obtain a copy of Informed Consent Form signed and dated. At the same time, I permit the State Food and Drug Administration/relevant state departments, Ethics Committees and relevant researchers to review the records.

Finally, I decide to participate the research.

Patient Signature:	______________	ID No.:	□□□□□□□□□□□□□□□□□□	
Contact number:	______________	Mobile phone number:	_____<br>_____<br>_____<br>_	Date:
Signature of legal representative:	______________	ID No.:	□□□□□□□□□□□□□□□□□□	
Contact number:	______________	Mobile phone number:	_____<br>_____<br>_____	Date:

_

I confirmed that I have explained the details of the trial to the patient, including his/her rights, potential profits and risks and provide him/her a copy of Informed Consent Form signed.

Physician Signature: ______________ Date:

Doctor's work telephone: ______________ Mobile phone number: ______________

Medical Ethics Committee Office of ***** Hospital, phone number: *********

Attachments: Record of Informed Consent Process

Record of Informed Consent Process

If the subject is unable to read or sign the informed consent form, the designated agent/guardian will record this page.

By what means does the subject give informed consent	☐ The doctor/nurse reads the contents of the informed consent form to the subject and answers the subject's questions.<br>☐ The agent reads the contents of the informed consent form to the subject and the doctor answers the subject's questions.<br>☐The subject's legal representative reads the subject's instructions and all questions have been answered by the doctor.<br>☐ Other: ______________________
Have the subjects understood all the contents of the instructions to the subjects	☐ Yes ☐ No ☐ Not applicable
Does the subject agree to participate in this trial	☐ Yes ☐ No ☐ Not applicable
Does the subject agree to all the stated terms on the "informed consent page"	☐ Yes ☐ No ☐ Not applicable
The reason why the subject was unable to sign this ICF	☐ Disability ☐ Illiterate ☐ Minor<br>☐ Other：______________________
The relationship between the agent and the subject	☐ Couple ☐ Parent ☐ Child ☐ Brother<br>☐ Other：______________________
Remarks:	

Signature of agent/guardian of subject:

Name (regular script)	Signature	Date of signature
		/ /
