# Supplementary material for: Zhizhu Kuanzhong Capsule in treating patients with functional dyspepsia postprandial distress syndrome: study protocol for a multicenter, randomized, double-blind, placebo-controlled, parallel-group clinical trial
Source: Trials. 2022 Jun 2;23:454. doi: 10.1186/s13063-022-06396-5 (PMC9161179; doi:10.1186/s13063-022-06396-5)
Supplement: Supplementary file 3 — Additional file 3. Names of all ethics committees and approval reference numbers of all participating institutions. [file 13063_2022_6396_MOESM3_ESM.doc]

| Number | Institution | Approval reference number |
| --- | --- | --- |
| China | | |
| 1 | Xiyuan Hospital of China Academy of Chinese Medical Sciences | 2018XLA074-4 |
| 2 | The First Affiliated Hospital of Sun Yat-sen University | 2019-171 |
| 3 | Peking Union Medical College Hospital of Chinese Academy of Medical Sciences | B334 |
| 4 | The First Affiliated Hospital of Guangzhou University of Chinese Medicine | ZYYECK-2020-069 |
| 5 | Jiangsu Province Hospital | 2019-SR-182.R1 |
| 6 | Ruijin Hospital | 2019-168 |
| 7 | Affiliated Hospital of Shanxi University of Traditional Chinese Medicine | 201908045 |
| 8 | Shaanxi Academy of Traditional Chinese Medicine | 2019-28 |
| 9 | Affiliated Hospital of Shaanxi University of traditional Chinese medicine | SZFYIEC-PJ-2019-18 |
| 10 | Renmin Hospital of Wuhan University | 2020K-Y007(C01) |
| 11 | Wuhan Union Hospital of China,Tongji Medical College,Huazhong University of Science and Technology | UHCT-IEC-SOP-016-02-01 |
| 12 | The Second Affiliated Hospital of Xi’an Jiaotong University | 2020-31 |
| 13 | Kulun Qimeng Hospital | 2018XLA074-4 |
| 14 | Hong Kong Baptist University | REC/19-20/0393 |
| 15 | Beijing Jishuitan Hospital | 202101-06 |
| 16 | Shengjing Hospital Affiliated to China Medical University | 2021PS464K |
| 17 | Fujian Provincial Hospital | 2021-007-01 |
| Australia | | |
| 18 | Princess Alexandra Hospital | HREC/2020/QMS/61038 |
